# Failure of a Highly Cross-Linked Polyethylene Liner After Spine Fusion

**DOI:** 10.5435/JAAOSGlobal-D-22-00150

**Published:** 2023-03-20

**Authors:** Kimberly Roesler, Kevin L. Garvin

**Affiliations:** From the Creighton University School of Medicine, Omaha, NE (Dr. Roesler), and the Department of Orthopaedic Surgery, University of Nebraska Medical Center, Omaha, NE (Dr. Garvin).

## Abstract

A 73-year-old woman, 11 years after total hip arthroplasty and 2 years after a multilevel lumbar spine fusion, presented with a 2-month history of anterior hip and gluteal pain. She was diagnosed with an acetabular liner fracture that occurred through the high wall, likely related to repetitive impingement on the neck of the femoral implant, as notable burnishing was observed on the explanted femoral head. The acetabulum was successfully revised to a dual-mobility articulation.

Spinal fusion after total hip arthroplasty can alter the acetabular implant position and was seen in our patient whose previously functional high-walled liner failed. Surgeons may consider alternative techniques, including a change in acetabular implant's anteversion to mitigate the need for a high-walled liner or the use of a dual-mobility bearing.

The presence of both degenerative spine disease and hip arthritis has been called hip-spine syndrome. Sing et al^1^ referred to hip-spine syndrome stating that this is likely increasing because of our aging population. The authors determined a statistically significant increase in complications for patients who had spinal arthrodesis and total hip arthroplasty (THA), compared with patients with THA alone. The risk of revision increased from 3.4% in the control group (THA only) to 7.8% in those patients with ≥3 levels of lumbar spine fusion.^1^ Other authors reported a higher risk of hip dislocation as a result of a stiff spine or impingement of the femoral implant against a high-walled liner.^2^ Duffy et al 2 reported failure of a high-walled polyethylene liner because of impingement; however, that case was not secondary to biomechanical changes in hip-spine relationship after spinal fusion.

A degree of increased spinal pelvic stiffness is part of the normal aging process.^3^ To maintain functional mobility, the hip compensates for a loss of spinal motion with an increase in femoral motion. It is well documented that 0.9° of femoral motion is gained for every 1° of spinal pelvic motion lost.^3,4,5,6,7^ Currently, there are no clear guidelines on how to treat a patient with concurrent spine and hip symptoms; however, it is generally recommended to treat the most symptomatic joint first.^4,5,8‐10^ If THA is done first, the spine disease-related stiffness can result in increased hip motion and instability or dislocation. While our case highlights posterior impingement, it should be noted that changing spinal alignment can result in a pelvis fixed more anteriorly or posteriorly. Changes in pelvic tilt contribute to changes in acetabular implant position and possible hip dislocation, potentially requiring revision surgery.^11^

Within this population, patients with a highly cross-lined polyethylene liner (HXLPE) and a high wall may also carry an increased risk of catastrophic failure because of the inability of the liner's thin high wall to withstand stress from repeated impingement. HXLPE was manufactured with the intention of decreasing the incidence of wear and debris-induced osteolysis after primary joint arthroplasty.^2,12^ Ultra-high–molecular-weight polyethylene is exposed to gamma radiation, which breaks up intramolecular bonds and produces free radicals.^2,12^ The free radicals promote polymer chain cross-linking and increase overall density.^2,12^ This material is thermally stabilized to prevent excess free radicals from causing oxidative instability.^2,12^

Fortunately, only a small subset of THA patients with a history of spinal stiffness or spinal degeneration requires a spine fusion. Inferior THA outcomes have been documented in patients before and after THA in the lumbar spinal fusion population.^9,13^ If a patient has a spinal fusion before THA, preoperative planning of the THA must account for restricted spinal mobility or the change in pelvic inclination, both of which require an appropriate implant position or the use of an implant with enhanced hip joint stability.^9^ Conversely, in our patient, spinal fusion and stiffness occurred after the primary THA altering the acetabular implant position, biomechanics, and stability of this patient's well-functioning THA.^14^

## Statement of Informed Consent

This patient was informed and consented to the data concerning the case being submitted for publication.

## Case Presentation

**Figure 1:**
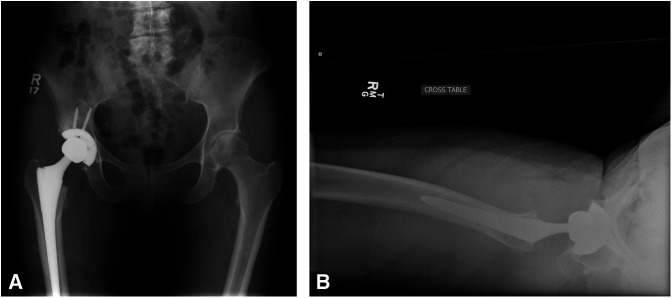
**A** and **B,** AP radiograph of the pelvis and lateral radiograph of the right hip.

A 73-year-old woman underwent THA 11 years before presentation. It should be noted that the high-walled liner was placed posterior and inferior, providing additional hip stability when the hip was flexed and adducted with slight hip internal rotation. No complications were documented during the primary THA or recovery. Postoperative radiographs demonstrated good position and alignment of the implants (Figure 1, A and B). The patient had excellent range of motion of her hip on recovery. She was able to ambulate without restrictions and denied pain 3 months postoperatively.

**Figure 2:**
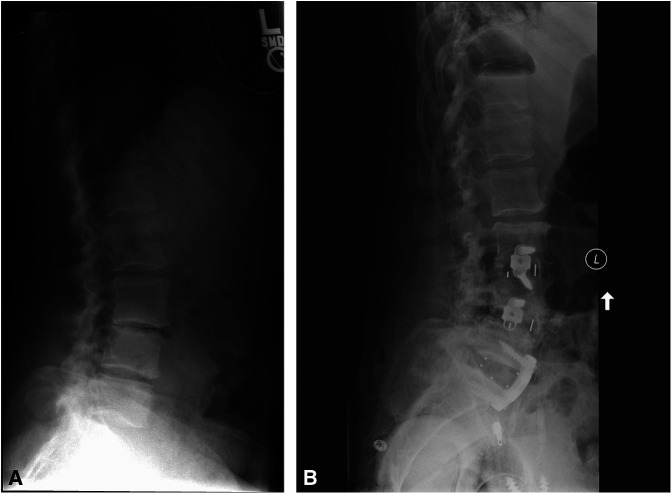
Lateral radiographs of the spine before the spinal fusion (**A**) and after a three-level spinal fusion conducted from L3 to the sacrum (**B**).

**Figure 3:**
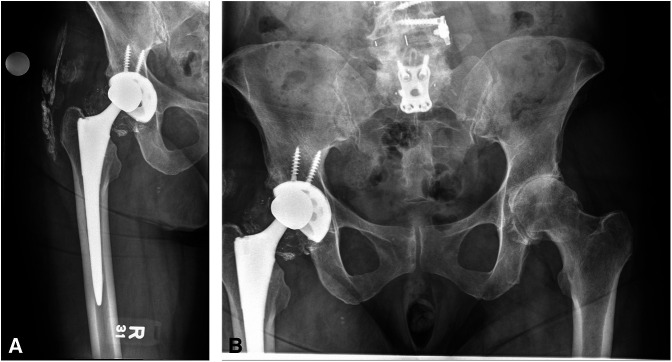
**A,** AP radiograph of the hip with an eccentric position of the femoral head and metallosis of the soft tissues. **B,** AP radiograph of the pelvis demonstrating the same changes.

**Figure 4:**
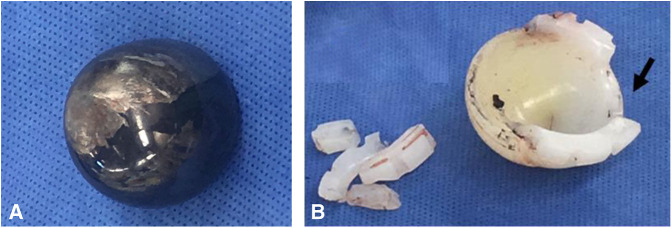
**A,** Image of the Oxinium head with abrasion or severe wear of the Oxinium (arrow) from the hip. **B,** Image of the retrieved acetabular liner with the linear fracture (arrow) along the high lip of the polyethylene acetabular implant and the polyethylene fragments from the high-all liner.

Six years after the THA, the patient had a single-level spinal fusion for long-standing symptoms of lumbar arthrosis and spinal stenosis. A second spinal fusion was completed three years later, 9 years after her THA. Approximately 2 years after her last spinal fusion (Figure 2, A and B), she presented with a 2-month history of pain over her anterior hip and the posterior gluteal region and a newly developed “grinding hip” sensation. Physical examination revealed a severe coxalgic gait with tenderness over the anterior hip capsule. The radiographs demonstrated an eccentric position of the femoral head with evidence of metallosis in the soft tissues (Figure 3, A and B). The pelvic radiograph demonstrates the flexed position of the pelvis secondary to the spine fusion. During the surgery, the acetabular liner was noted to be fractured into several pieces and the Oxinium femoral head was significantly worn with metal debris from the head and acetabular implant throughout the soft tissues (Figure 4, A and B). The diagnosis was polyethylene failure and metallosis.

**Figure 5:**
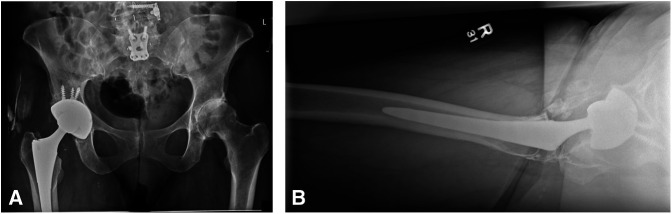
**A,** Postoperative AP pelvis radiograph after revision surgery showing the dual-mobility implants and (**B**) the postoperative lateral radiograph.

The damaged tissue and metallosis was excised and the acetabular implant revised to a new dual-mobility acetabular implant. The new implant was revised with a more anteverted position compared with the previous acetabular implant. This change in anteversion was accomplished by marking the anteversion of the first implant and then placing the second implant in increased anteversion based on the markings of the new dual-mobility acetabular liner. Surgery was completed without complication, and radiographs showed good implant positioning (Figure 5, A and B). The patient has been followed for 2 years and resumed her previous activity level now, 2 years after her revision.

## Discussion

In this case, the femoral and acetabular implants were initially placed based on the degree of hip arthritis and spinal alignment at the time of surgery. Subsequent progression of spinal disease and the need for fusion of the spine could not have been predicted at the time of the patient's primary THA. Thus, it is understandable that notable loss of spine motion, because of the spine fusion, affected the biomechanics of the previously placed THA. The comprehensive effects of altered biomechanics of the hip resulting from the spine fusion, ultimately contributing to the acetabular polyethylene liner failure, are less well-documented in patients with concurrent spine fusion and THA.

As previously mentioned, HXPLE is engineered with the intention of improving wear characteristics; however, the radiation and subsequent remelting results in theoretically less fatigue resistance of the HXPLE.^4^ While no studies were found that reported an increased incidence of linear fractures since the introduction of HXPLE, excess force placed on the rim because of cup alignment increases risk of rim cracking.^15^ Tower et al ^15^ reported failure of four Longevity cross-linked polyethylene acetabular liners related to thinner polyethylene at the cup rim, vertical cup alignment, and mechanical properties. In accordance with Tower et al, the likely cause of failure of the Longevity acetabular implant in the presented case was repetitive femoral neck impingement resulting in a fatigue fracture of the extended liner beyond the acetabular cup.

Similar to the case presented, Duffy et al^2^ described failure of an extended lip liner (Marathon cross-linked polyethylene by DePuy). In our patient, failure 11 years after THA and 2 years after multilevel spine fusion because of impingement-induced fatigue, failure of the extended lip occurred while the hip was fully extended.^4^ The rim of the liner subsequently fractured, leading to polyethylene dissociation.^16^ It should be noted that failure in the presented case occurred in a HXPLE (Longevity, Zimmer Biomet) 2 years after spinal fusion. Although the time lines are slightly different, the basic failure etiology is the same. The increased spinal stiffness due to arthritis and subsequent fusion altered the hip range of motion, causing impingement and catastrophic failure. Intraoperative findings included fracture of the liner rim, which prevented the liner's stability within the acetabular implant.

The combination of impingement of the femoral neck on a high-walled liner because of the spinal fusion combined with the decreased fatigue resistance of the HXPLE put our patient at risk of catastrophic polyethylene failure. In particular, the high wall of the polyethylene liner implanted in our patient's case was placed under greater strain after her spinal fusion, leading to failure.

Finally, this patient's problem may question the use of a high-walled liner for THA patients. The high wall provides protection from hip dislocation in one direction, but it risks femoral neck impingement in the opposite direction. In this patient, the high-walled liner was placed posterior and inferior providing additional hip stability when the hip was flexed and adducted with slight hip internal rotation. However, with the hip extended and externally rotated, the neck of the femoral implant impinged against the high wall and, over time, repetitive impingement caused a fracture of the high-walled area. Retrospectively, we propose it may have been better for our patient to antevert the original acetabular implant slightly more and avoid the need for a high-walled liner.

In summation, this case report describes a patient who developed late failure of the hip and polyethylene liner after spinal fusion. Spinal fusion after THA increases the risk of hip dislocation because of changes in acetabular implant position and the documented increase in femoral motion. Within this subset of THA patients, those who have HXLPE and a high-walled liner are known to have a risk of failure secondary to fatigue resistance compared with those with a non-cross linked polyethylene liner. In patients with concurrent spine and hip arthritis, it is important to consider possible conflicts in the biomechanics of the current implant and elevated polyethylene liners that may possibly be avoided.^1,2,3,4,5,8,17,18,19,20,21^
